# The impact of ossification spread on cervical spine function in patients with ossification of the posterior longitudinal ligament

**DOI:** 10.1038/s41598-021-93602-0

**Published:** 2021-07-12

**Authors:** Keiichi Katsumi, Takashi Hirai, Toshitaka Yoshii, Satoshi Maki, Kanji Mori, Narihito Nagoshi, Soraya Nishimura, Kazuhiro Takeuchi, Shuta Ushio, Takeo Furuya, Kei Watanabe, Norihiro Nishida, Kota Watanabe, Takashi Kaito, Satoshi Kato, Katsuya Nagashima, Masao Koda, Kenyu Ito, Shiro Imagama, Yuji Matsuoka, Kanichiro Wada, Atsushi Kimura, Tetsuro Ohba, Hiroyuki Katoh, Yukihiro Matsuyama, Hiroshi Ozawa, Hirotaka Haro, Katsushi Takeshita, Masahiko Watanabe, Morio Matsumoto, Masaya Nakamura, Masashi Yamazaki, Atsushi Okawa, Yoshiharu Kawaguchi

**Affiliations:** 1 Spine Center, Department of Orthopedic Surgery, Niigata Central Hospital, 1-18 Shinkocho, Chuo-ku, Niigata, Niigata 950-8556 Japan; 2 Department of Orthopedic Surgery, Niigata University Medical and Dental General Hospital, 1-757 Asahimachidori, Chuo-ku, Niigata, Niigata 951-8510 Japan; 3 Department of Orthopedic Surgery, Tokyo Medical and Dental University, 1-5-45 Yushima, Bunkyo-ku, Tokyo 113-8519 Japan; 4 Department of Orthopaedic Surgery, Chiba University Graduate School of Medicine, 1-8-1 Inohana, Chuo-ku, Chiba, Chiba 260-0856 Japan; 5 Department of Orthopaedic Surgery, Shiga University of Medical Science, Tsukinowa-cho, Seta, Otsu, Shiga 520-2192 Japan; 6 Department of Orthopaedic Surgery, Keio University, School of Medicine, 35 Shinanomachi, Shinjuku-ku, Tokyo Japan; 7 Department of Orthopedic Surgery, National Hospital Organization Okayama Medical Center, 1711–1 Tamasu, Okayama, Okayama Japan; 8 Department of Orthopaedic Surgery, Yamaguchi University Graduate School of Medicine, 1-1-1 Minamikogushi, Ube, Yamaguchi 755-8505 Japan; 9 Department of Orthopaedic Surgery, Osaka University Graduate School of Medicine, 2-2 Yamadaoka, Suita, Osaka 565-0871 Japan; 10 Department of Orthopedic Surgery, Graduate School of Medical Sciences, Kanazawa University, 13-1, Takaramachi, Kanazawa, Ishikawa 920-8641 Japan; 11 Department of Orthopaedic Surgery, Faculty of Medicine, University of Tsukuba, 2-1-1 Amakubo, Tsukuba, Ibaraki 305-8576 Japan; 12 Department of Orthopedic Surgery, Nagoya University Graduate School of Medicine, 65 Tsurumaicho, Showa-ku, Nagoya, Aichi 466-0065 Japan; 13 Department of Orthopedic Surgery, Tokyo Medical University, 6-7-1 Nishishinjuku, Shinjuku-ku, Tokyo 160-0023 Japan; 14 Department of Orthopaedic Surgery, Hirosaki University Graduate School of Medicine, 53 Honcho, Hirosaki, Aomori 036-8203 Japan; 15 Department of Orthopaedic Surgery, Jichi Medical University, 3311-1 Yakushiji, Shimotsuke, Tochigi 329-0498 Japan; 16 Department of Orthopedic Surgery, University of Yamanashi, 1110 Shimokato, Chuo-ku, Yamanashi 409-3898 Japan; 17 Department of Orthopaedic Surgery, Surgical Science, Tokai University School of Medicine, 143 Shimokasuya, Isehara, Kanagawa 259-1143 Japan; 18 Department of Orthopedic Surgery, Hamamatsu University School of Medicine, 1-20-1 Handayama, Hamamatsu, Shizuoka 431-3125 Japan; 19 Department of Orthopaedic Surgery, Tohoku Medical and Pharmaceutical University, 1-12-1 Fukumuro, Miyagino-ku, Sendai, Miyagi 983-8512 Japan; 20 Department of Orthopedic Surgery, Faculty of Medicine, University of Toyama, 2630 Sugitani, Toyama, Toyama 930-0194 Japan; 21 Japanese Organization of the Study for Ossification of Spinal Ligament (JOSL), Tokyo, Japan

**Keywords:** Neuropathic pain, Spinal cord diseases, Physical examination, Bone imaging, Radiography, Whole body imaging, Computed tomography, Quality of life, Risk factors, Pain, Disability

## Abstract

Ossification of the posterior longitudinal ligament (OPLL) is a progressive disease. The bridging of ossified lesions to the vertebral body gradually increases, thereby decreasing the mobility of the cervical spine; thus, cervical spine function may decrease over time. However, cervical spine function in patients with cervical OPLL has not been evaluated in large prospective studies. Therefore, we conducted a prospective multicenter study to clarify whether ossification spread can influence cervical spine function and quality of life (QOL) in patients with cervical OPLL. In total, 238 patients (162 men, 76 women; mean age, 63.9 years) were enrolled from 16 institutions. Each patient underwent whole spine computed tomography and was evaluated for cervical spine function and QOL using the Japanese Orthopaedic Association Cervical Myelopathy Evaluation Questionnaire (JOACMEQ). In the multivariate regression analysis, a higher neck VAS score and a larger number of bridge formations of OPLL in the whole spine were significant predictors of adverse outcomes related to cervical spine function. This is the first prospective multicenter study to reveal the impact of ossification spread on cervical spine function. These findings are important to understand the natural course of OPLL and can serve as controls when evaluating postoperative cervical spine function.

## Introduction

Ossification of the posterior longitudinal ligament (OPLL) was first described by Key in 1838^1^, and the concept of OPLL has been widely used since Tsukimoto published an autopsy case report on this subject in 1960^2^. OPLL is characterized by the replacement of ligamentous tissue by ectopic bone formation and has been recognized as one of the main causes of cervical myelopathy^2–4^. Although several reports have demonstrated the pathophysiology of OPLL, its clinical features, and outcome data of surgical treatments^3–5^, there are few reports on cervical spine function in patients with OPLL. Because OPLL is a progressive disease, the bridging of ossified lesions to the vertebral body gradually increases, thereby decreasing the mobility of the cervical spine; thus, cervical spine function and quality of life (QOL) may decrease over time. Understanding cervical spine function in patients with OPLL who had been treated conservatively is important in evaluating the natural history of OPLL and the side effects of surgical treatment, in particular, of posterior surgery.

Although the Japanese Orthopaedic Association (JOA) score^6^ is used as a functional assessment of cervical myelopathy worldwide, the scoring system does not include cervical spine function and neck pain, both of which seriously impact patients’ QOL (Supplemental Table 1). In 2007, the JOA established a self-administered questionnaire, the Japanese Orthopaedic Association Cervical Myelopathy Evaluation Questionnaire (JOACMEQ), as a new outcome measurement tool for patients with cervical myelopathy, which included measurements of cervical spine function such as neck pain, stiff neck, disability, and QOL (Supplemental Table 2)^7^.

**Table 1:** The domain of cervical spine function in the JOACMEQ.

	Questionnaire	Score
Q1-1	While in the sitting position, can you look up at the ceiling by tilting your head upward?	
	1) Impossible	1
	2) Possible to some degree (with some effort)	2
	3) Possible without difficulty	3
Q1-2	Can you drink a glass of water without stopping despite your neck symptoms?	
	1) Impossible	1
	2) Possible to some degree	2
	3) Possible without difficulty.	3
Q1-3	While in the sitting position, can you turn your head toward a person who is seated to the side but behind you and speak to that person while looking at his/her face?	
	1) Impossible	1
	2) Possible to some degree	2
	3) Possible without difficulty	3
Q1-4	Can you look at your feet when you go downstairs?	
	1) Impossible	1
	2) Possible to some degree	2
	3) Possible without difficulty	3

*JOACMEQ* Japanese Orthopaedic Association Cervical Myelopathy Evaluation Questionnaire.

The score for the cervical spine function domain was calculated as follows (points): Q1-1 × 20 + Q1-2 × 10 + Q1-3 × 15 + Q1-4 × 5 − 50.

In this study, we conducted a prospective multicenter study of patients with cervical OPLL who had been treated conservatively to evaluate the existence and distribution of OPLL in the whole spine using computed tomography (CT). Ossification spread in the whole spine is represented by the OPLL ossification index (OP-index), which was defined as the sum of the levels of ossification at the vertebral bodies and the intervertebral discs^8–10^. Similarly, when the ossified lesions formed a bridge with the posterior border of the adjacent vertebral body, it was judged as “bridge formation”^9^ .We examined the bridge formations of OPLL in the whole spine. Furthermore, we investigated cervical spine function and QOL using the JOACMEQ. The purpose of this study was to clarify whether a higher OP-index and/or a larger number of bridge formations of OPLL can influence cervical spine function and QOL in patients with cervical OPLL.

## Materials and methods

This prospective multicenter study was conducted at the 16 institutions of the Japanese Multicenter Research Organization for Ossification of the Spinal Ligament established by the Japan Ministry of Health, Labour and Welfare. This study was approved by the institutional review board of each institution and performed in accordance with approved guidelines and in compliance with the principles of the Declaration of Helsinki. Informed consent was obtained from all patients before enrollment. This study included non-surgically treated patients with cervical OPLL diagnosed by neck radiographs who had symptoms such as neck pain, numbness in the upper or lower extremities, clumsiness, or gait disturbance. The conservative treatment included close observation and/or pain management medication. Patients who had a history of previous cervical surgeries and were younger than 20 years were excluded. Each patient underwent whole spine CT and was evaluated based on the JOACMEQ. A total of 238 patients (162 men, 76 women; mean age, 63.9 years; range, 36–92 years) were enrolled prospectively from September 2015 to December 2017.

Cervical spine function and radiological examinations including the extent of ossified lesions obtained from CT images of the whole spine in conservatively treated OPLL patients were conducted. In addition, we examined the effects of the spread of ossification on cervical spine function.

### Clinical examinations

Age, sex, height, weight, body mass index (BMI), and presence of diabetes mellitus (DM) were recorded as basic clinical data. The clinical status was evaluated using the JOA score and JOACMEQ. The JOA score is a 17-point instrument where points are assigned based on the ratings of motor function, sensory function, and urinary bladder function that is used as a functional assessment of cervical myelopathy worldwide. The JOACMEQ, which is a self-administered questionnaire, includes 24 questions corresponding to five domains: Q1 (cervical spine function), Q2 (upper extremity function), Q3 (lower extremity function), Q4 (bladder function), and Q5 (quality of life). The score of each domain ranges from 0 to 100 and is proportional to the patients’ clinical conditions; a normal score is 100 points. Cervical spine function was evaluated using four questions: Q1-1 and Q1-2 (neck extension), Q1-3 (neck rotation), and Q1-4 (neck flexion) from domain Q1 of the JOACMEQ (Table 1). The score for the cervical spine function domain was calculated as follows (points): Q1-1 × 20 + Q1-2 × 10 + Q1-3 × 15 + Q1-4 × 5 − 50. The 100-mm visual analog scale (VAS) was used to evaluate the degree of pain or stiffness in the neck or shoulders.

### Radiological examinations

**Figure 1:**
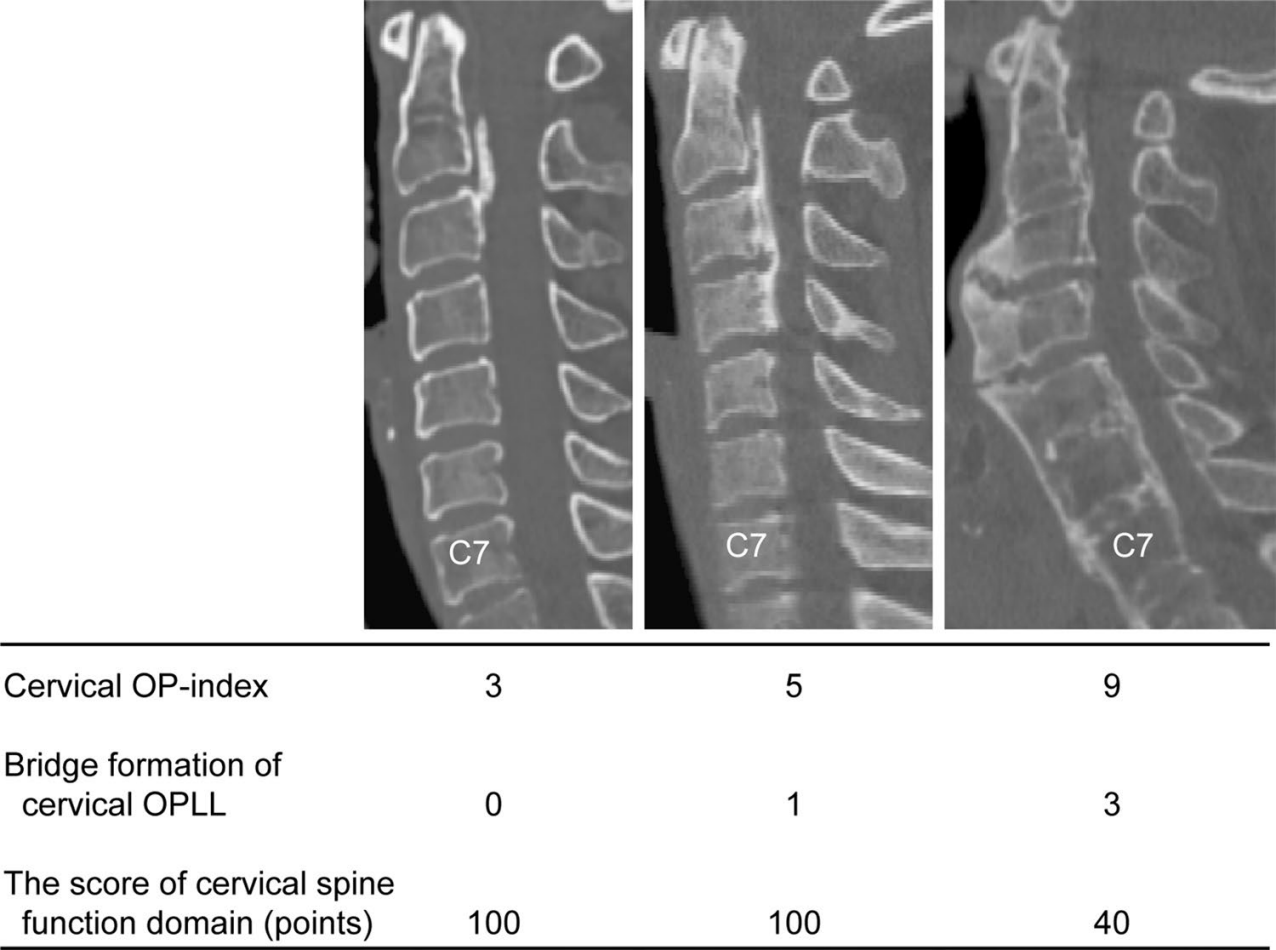
Representative reconstructive mid-sagittal computed tomography image of cervical OP-index and bridge formation of cervical OPLL. The score of the cervical spine function domains of the left, middle, and right images were 100 points, 100 points, and 40 points, respectively. *OPLL* ossification of the posterior longitudinal ligament; *OP-index* ossification index for OPLL.

The OPLL incidence in the whole spine from the clivus to S1 was evaluated using mid-sagittal CT images. Observed ossified lesions were recorded for each vertebral body and at the intervertebral disc level. We recorded the OP-index not only for the cervical spine (0–14; C1, C1/C2…C7, C7/T1) but also for the whole spine (0–49; C1… S1). Using a previously reported method^8^, we categorized patients into three groups according to the cervical OP-index: Grade 1 (cervical OP-index, ≤ 5), Grade 2 (cervical OP-index, 6–9), and Grade 3 (cervical OP-index, ≥ 10). Furthermore, we examined the bridge formation of OPLL from the cervical spine (0–7; C1/C2…C7/T1) to the whole spine (0–24; C1/C2…L5/S1) (Fig. 1). All CT data were evaluated by six experienced spine surgeons (KK, KM, SM, NN, SU, and KT). Before the analysis, all observers read the same CT images of 20 patients to confirm interobserver intra-class correlation coefficients (ICCs). The mean interobserver ICC was 0.83 (0.79–0.85), indicating a substantial agreement and consistency with previous results^8^.

### Statistical analyses

Data were expressed as mean and standard deviations. Differences between groups were evaluated using Mann–Whitney U-tests. Kruskal–Wallis analysis was applied to compare the four questions of the cervical spine function domain in the JOACMEQ, the three groups according to cervical OP-index grade, and the five different age groups. Spearman’s rank correlation coefficients between the clinical or radiological findings and cervical spine function were calculated. We categorized patients into two groups based on points for cervical spine function: good cervical function (≥ 50 points) and poor cervical function (< 50 points), and multiple logistic regression analysis was performed to identify variables independently associated with cervical spine function. First, the significance of the variables was evaluated using univariate analysis. Next, variables with *p* < 0.1 were subjected to multivariate analysis and *p* < 0.05 was considered significant. Statistical analyses were performed using SPSS software (version 25; IBM Corp., Armonk, NY, USA).

### Ethics approval and consent to participate

Written informed consent was obtained from each study participant before enrollment at each institution. The study protocol was approved by the ethics committee at each participating institution i.e., Niigata Central Hospital: et2020-03, Niigata University Medical and Dental General Hospital: 2015-2525, Tokyo Medical and Dental University: M2000-2068, Chiba University Graduate School of Medicine: 1654, Shiga University of Medical Science: 25-108, Keio University: 20180096, National Hospital Organization Okayama Medical Center: H28-71, Yamaguchi University Graduate School of Medicine: H27-117, Osaka University Graduate School of Medicine: 15153-2, Kanazawa University: 2015-087, University of Tsukuba: H26-108, Nagoya University Graduate School of Medicine: 2005-0354, Hirosaki University Graduate School of Medicine: 2015-209, Jichi Medical University: A17-106, University of Yamanashi: 1239, Tokai University School of Medicine: 13R-297, Hamamatsu University School of Medicine: 15-279, Tohoku Medical and Pharmaceutical University: 2016-2-041, University of Toyama, Toyama: 25-42.

## Results

### Clinical examinations

**Figure 2:**
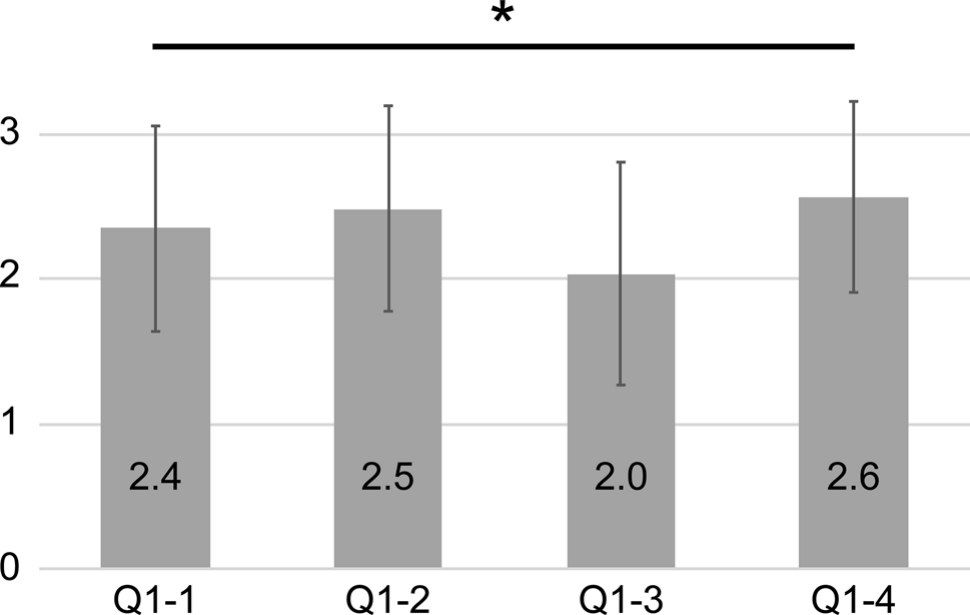
The scores for each question of Q1-1 and Q1-2 (neck extension), Q1-3 (neck rotation), and Q1-4 (neck flexion). The score of Q1-3 was significantly lower than that of the other questions. *p < 0.05.

Height, weight, and BMI were 162.7 ± 9.7 cm (range, 138–187 cm), 69.1 ± 15.2 kg (range, 32–114 kg), and 25.9 ± 4.5 kg/m^2^ (range, 16.4–45.2 kg/m^2^), respectively. Fifty-nine of the 238 patients (24.8%) had DM, and the cervical JOA score was 12.3 ± 3.4 points (range, -2–17 points). The prevalence of neck pain was 60.0% (142/238 patients), and the neck or shoulder VAS score was 38.8 ± 31.3 (range, 0–100). The scores for each question of Q1-1, Q1-2, Q1-3, and Q1-4 were 2.4 ± 0.7, 2.5 ± 0.7, 2.0 ± 0.8, and 2.6 ± 0.7, respectively (Fig. 2). The score for Q1-3 was significantly lower than that of the other questions (*p* < 0.05). The score for the domain of cervical spine function was 65.9 ± 28.5 points. According to the cervical OP-index grading, the scores of cervical spine function were 67.8 ± 27.1 in Grade 1, 66.8 ± 29.4 in Grade 2, and 57.0 ± 30.4 in Grade 3. There were no significant differences between the cervical OP-index grading groups. The scores for the Q2, Q3, Q4, and Q5 domains were 80.1 ± 21.6, 65.9 ± 30.8, 74.4 ± 22.1, and 49.8 ± 19.9, respectively. The cervical spine function domain (Q1) score was positively correlated with the Q2 (r = 0.47, *p* < 0.001), Q3 (r = 0.41, *p* < 0.001), Q4 (r = 0.35, *p* < 0.001), and Q5 (r = 0.45, *p* < 0.001) domain scores.

### Radiological examinations

The OP-index of the cervical spine and whole spine was 6.0 ± 3.2 (range, 1–14) and 8.6 ± 6.4 (range, 1–38), respectively. The cervical OP-index was Grade 1 in 116 patients (48.7%), Grade 2 in 87 patients (36.6%), and Grade 3 in 35 patients (14.7%). The number of bridge formations of OPLL in the cervical spine and the whole spine were 0.7 ± 1.1 (range, 0–6) and 1.4 ± 0.3 (range, 0–17), respectively.

### Sex difference and age-related change

The OP-index and bridge formations of OPLL for the whole spine of women were significantly higher than those of men (both *p* < 0.05) (Table 2). Patients were divided into five groups based on age categories: ≤ 49 years (n = 37), 50–59 years (n = 48), 60–69 years (n = 66), 70–79 years (n = 71), and ≥ 80 years (n = 16). Because of the small number of patients, four men and one woman in their 30 s and one man and one woman in their 90 s were included in the groups of ≤ 49 years and ≥ 80 years, respectively. Cervical spine function was significantly lower in the 70–79-years and ≥ 80-years age groups than in the ≤ 49-years age group (both *p* < 0.05). In the questions of the cervical spine function domain, the score for each question tended to decline with age; however, only Q1-2 showed a significant difference (*p* < 0.05). Moreover, the score of Q1-3 was significantly lower than those of Q1-2 and Q1-4 in all age groups except in the ≥ 80-years age group (all *p* < 0.05). Bridge formation of OPLL in the cervical spine was significantly higher in the ≥ 80-years age group than in the ≤ 49-years and 50–59-years age groups (both *p* < 0.05) (Table 3).

**Table 2:** Sex differences in parameters.

Characteristic	Male (n = 162)	Female (n = 76)	*p* value
Age (years)	64.0 ± 12.7	63.6 ± 11.5	0.51
BMI (kg/m^2^)	25.9 ± 4.3	26.1 ± 4.8	0.90
JOA score (points)	12.0 ± 3.5	12.8 ± 3.1	0.19
Neck VAS score	38.8 ± 32.3	38.8 ± 29.2	0.78
Cervical spine function (points)	65.1 ± 29.4	65.9 ± 27.5	0.96
**Questions of Q1 domain**			
Q1-1	2.3 ± 0.7	2.4 ± 0.7	0.25
Q1-2	2.5 ± 0.7	2.5 ± 0.7	0.61
Q1-3	2.1 ± 0.8	2.0 ± 0.8	0.83
Q1-4	2.6 ± 0.6	2.5 ± 0.8	0.09
C-OP-index	6.0 ± 3.1	6.0 ± 3.4	0.81
W-OP-index	7.5 ± 5.1	10.9 ± 8.1	0.006**
Cervical OP-index grade	1.7 ± 0.7	1.6 ± 0.8	0.44
Bridge formation of C-OPLL	0.7 ± 1.2	0.7 ± 1.1	0.76
Bridge formation of W-OPLL	1.1 ± 1.8	2.0 ± 3.0	0.014*

Data are presented as mean ± standard deviation.

*BMI* body mass index; *JOA* Japanese Orthopaedic Association; *VAS* visual analog scale (100-mm). Q1 domain, cervical spine function domain of the Japanese Orthopaedic Association Cervical Myelopathy Evaluation Questionnaire; *C*, cervical; *W*, whole spine; *OP-index* Ossification index for OPLL; bridge formation, ossified lesions forming a bridge with the posterior border of the adjacent vertebral body.

*p < 0.05; **p < 0.01.

**Table 3:** Age-related changes in parameters.

Characteristic	≤ 49 years (n = 37)	50–59 years (n = 48)	60–69 years (n = 66)	70–79 years (n = 71)	≥ 80 years (n = 16)	*p* value
Age (years)	43.8 ± 3.5	54.8 ± 2.8	65.4 ± 2.8	74.6 ± 2.7	84.3 ± 3.3	0.000***
Male sex, no. (%)	26 (70.3)	32(66.7)	42(63.6)	50(70.4)	12(75.0)	0.87
BMI (kg/m^2^)	28.9 ± 5.4	27.4 ± 4.3	25.7 ± 3.9	24.5 ± 3.6	22.2 ± 2.6	0.000***
JOA score (points)	12.8 ± 2.7	12.5 ± 3.0	12.8 ± 3.4	12.0 ± 3.4	9.6 ± 5.0	0.07
Neck VAS score	40.5 ± 32.5	41.2 ± 31.5	32.0 ± 28.2	41.0 ± 31.9	46.1 ± 31.9	0.34
Cervical spine function (points)	78.2 ± 23.3	67.4 ± 28.8	67.1 ± 27.7	58.2 ± 30.8	54.4 ± 24.8	0.005**
**Questions of Q1 domain**						
Q1-1	2.7 ± 0.5	2.2 ± 0.8	2.4 ± 0.7	2.3 ± 0.7^a^	2.2 ± 0.7	0.07
Q1-2	2.8 ± 0.6^a^	2.7 ± 0.6^a^	2.6 ± 0.6^a^	2.3 ± 0.8^a^	2.2 ± 0.8	0.001**
Q1-3	2.3 ± 0.7^a^	2.1 ± 0.8^a^	2.1 ± 0.8^a^	1.9 ± 0.8^a^	1.8 ± 0.7	0.07
Q1-4	2.7 ± 0.6^a^	2.7 ± 0.5^a^	2.6 ± 0.6^a^	2.4 ± 0.8^a^	2.3 ± 0.8	0.11
C-OP-index	5.4 ± 3.2	5.8 ± 3.2	5.9 ± 3.0	6.1 ± 3.2	7.5 ± 3.4	0.33
W-OP-index	9.4 ± 7.7	9.1 ± 6.3	7.5 ± 5.0	8.7 ± 7.0	9.5 ± 5.4	0.70
Bridge formation of C-OPLL	0.5 ± 0.7	0.5 ± 1.0	0.8 ± 1.2	0.8 ± 1.2	1.5 ± 1.6	0.028*
Bridge formation of W-OPLL	1.5 ± 2.3	1.1 ± 1.9	1.3 ± 1.8	1.6 ± 2.9	1.8 ± 1.9	0.36

Data are presented as mean ± standard deviation.

*BMI* body mass index; *JOA* Japanese Orthopaedic Association; *VAS* visual analog scale (100-mm).

*Q1 domain* cervical spine function domain of the Japanese Orthopaedic Association Cervical Myelopathy Evaluation Questionnaire; *C*, cervical; *W*, whole spine; *OP-index* ossification index for OPLL; *bridge formation* ossified lesions forming a bridge with the posterior border of the adjacent vertebral body.

^a^Significant differences were found among the questions in each age group (all *p* < 0.05).

*p < 0.05; **p < 0.01; ***p < 0.001.

### Correlation between cervical spine function and other clinical and radiological parameters

Cervical spine function was negatively correlated with age and neck VAS (r =  − 0.23, *p* < 0.001; r =  − 0.45, *p* < 0.001; respectively) and positively correlated with the JOA score (r = 0.31, *p* < 0.001). In the radiological findings, there was a negative correlation between cervical spine function and bridge formation of OPLL in the whole spine (r =  − 0.15, *p* < 0.05).

### Logistic regression analysis of factors adversely associated with cervical spine function

The univariate analysis demonstrated that there were significant correlations with age, JOA score, neck VAS, cervical OP-index grade, OP-index, and bridge formation of OPLL in the cervical and whole spine (all *p* < 0.05) (Table 4). In the multivariate regression analysis, a higher neck VAS score (odds ratio [OR] = 0.978, 95% confidence interval [CI] = 0.967–0.988, *p* = 0.000) and a larger number of bridge formations of OPLL in the whole spine (OR = 0.820, 95% CI = 0.713–0.943, *p* = 0.005) were independent factors that adversely affected cervical spine function.

**Table 4:** Comparison between the groups with poor and good cervical function.

Characteristic	Poor cervical function group (n = 62)	Good cervical function group (n = 176)	*p* value
Age (years)	66.9 ± 11.2	62.8 ± 12.5	0.024*
Male sex, no. (%)	44 (71.0)	118 (67.1)	0.57
Body height	162.6 ± 9.4	162.7 ± 9.9	0.90
Body weight	69.5 ± 16.4	68.9 ± 14.8	0.79
BMI	26.1 ± 4.9	25.9 ± 4.3	0.70
Comorbid diabetes mellitus, no. (%)	17 (27.4)	42 (23.9)	0.58
JOA score	11.1 ± 3.7	12.7 ± 3.2	0.001**
Neck VAS score	55.1 ± 32.4	33.1 ± 28.8	0.000***
C-OP-index	6.8 ± 3.4	5.7 ± 3.1	0.018*
W-OP-index	10.0 ± 7.8	8.1 ± 5.7	0.046*
Cervical OP-index grade	1.8 ± 0.8	1.6 ± 0.7	0.024*
Bridge formation of C-OPLL	1.1 ± 1.4	0.6 ± 1.0	0.010*
Bridge formation of W-OPLL	2.2 ± 3.2	1.1 ± 1.8	0.003**

Data are presented as mean ± standard deviation.

*BMI* body mass index; *JOA* Japanese Orthopaedic Association; *VAS* visual analog scale (100-mm); *C*, cervical; *W*, whole spine; *OP-index* ossification index for OPLL; *bridge formation* ossified lesions forming a bridge with the posterior border of the adjacent vertebral body.

*p < 0.05; **p < 0.01; ***p < 0.001.

## Discussion

The analysis of treatment outcomes using patient-based assessments is becoming standard practice. The JOACMEQ is a patient-based assessment for evaluating cervical compressive myelopathy, and several reports using this scoring system have been published to date^7,11–14^. Regarding the QOL of patients with OPLL, several reports on postoperative axial neck pain have been published^12,15,16^; however, to the best of our knowledge, detailed reports of cervical spine function based on large prospective multicenter series are lacking. In this study, the cervical spine function domain score was positively correlated with the QOL domain score, indicating that cervical spine function can influence the QOL of patients with OPLL. Comprehensive evaluations of cervical spine function in patients with OPLL who underwent conservative treatment are important to understand the natural course of OPLL and can serve as a control when evaluating cervical spine function after surgery.

In this study, the score of cervical spine function was 65.9 points (age, 63.9 years; JOA score, 12.3 points). Tanaka et al.^17^ examined the standard value of the JOACMEQ in 1629 healthy Japanese volunteers and reported that the score of cervical spine function was > 90 points in individuals in their 20 s to 60 s, whereas the score decreased to 80 and 70 points, respectively, in those in their 70 s and 80 s. Moreover, Ohya et al.^13^ reported that cervical spine function in 44 patients with OPLL (age, 63.8 years; preoperative JOA score, 10.9 points) was 55.5 points preoperatively and 64.9 points postoperatively. Our results were also lower than those of the healthy volunteers reported by Tanaka et al.^17^ and similar to the findings of Ohya et al.^13^ Even in the patients aged ≤ 49 years, which was the group with the highest points in this study, their points were equivalent to those of healthy volunteers in their 80 s as reported by Tanaka et al. In the present study, the score of Q1-3 was significantly lower than that of the other questions in the overall cohort and many age groups. In general, the range of motion (ROM) in the cervical spine decreases due to age-related and degenerative changes; however, flexion movement is considered to be less affected than extension movement^18,19^. Yuan et al.^20^ reported that patients with cervical OPLL had decreased cervical ROM including flexion, extension, and rotation compared to the normal controls. Our study revealed that rotation movement was the most limited, and flexion and extension movements were generally comparable. Whether this result is due to a decrease in ROM, neck pain, or neurologic compression remains to be solved.

This study also investigated age-related changes in parameters and found that cervical spine function tended to decline with age from middle age. Since the neck VAS scores did not differ significantly by age, we considered that a decline in cervical spine function was caused by the decreasing mobility of the cervical spine. As several studies have demonstrated the progression of ossification in many patients with OPLL^3–5,21^, we considered that the OP-index and bridge formation of OPLL increased with age. However, although there appeared to be an increase in the tendency of bridge formations with age, a significant difference was found only in the bridge formations in the cervical spine. Thus, we speculated that factors other than age may be involved in the onset and progression of OPLL.

In the correlation analysis, age, neck VAS, and the JOA score were significantly correlated with cervical spine function. In addition, bridge formation of OPLL was significantly correlated with the whole spine but not with the cervical spine. Furthermore, following logistic regression analysis, only two independent factors were significantly associated with adverse cervical spine function namely, a higher neck VAS score and a larger number of bridge formations of OPLL in the whole spine.

From the results of this study, neck pain caused by degenerative changes and/or neurologic deficits had a greater effect on cervical spine function in the clinical findings. Furthermore, the radiological findings revealed that the spread of bridge formations of the vertebrae, rather than that of ossified lesions at the vertebra and intervertebral discs, and in the whole spine, rather than in the cervical spine, had a greater effect on cervical spine function. Fujimori et al.^22^ evaluated the intervertebral segmental ROM in patients with OPLL at the intervertebral discs between bridging type (ossification bridging intervertebral segment with bony union) and non-bridging type using functional CT and reported that the intervertebral ROMs were 0.3° in the bridging type and 4.9° in the non-bridging type. We consider that the structural impairment caused by bridge formation of OPLL has an impact on the deterioration of cervical spine function. According to previous reports, cervical alignment is influenced by the global spine alignment, and concomitant thoracic spine mobility is necessary to produce the complete range of movements at the cervical spine^23,24^. Reduced mobility in the thoracic spine has also been reported to cause neck pain and dysfunction^25^. Therefore, when assessing the impact of OPLL on cervical spine function, we believe it is important to pay attention to bridge formation of OPLL, not only in the cervical spine but also in the whole spine. In summary, cervical spine dysfunction can be divided into two categories namely, functional impairment due to pain radiating to the neck and shoulders and structural impairment due to decreased mobility of the whole spine caused by bridge formations of ossified lesions spreading to the vertebral body. It is clinically important to recognize these impairments when evaluating cervical spine function in patients with OPLL.

This study has several limitations. First, although this study was a prospective nationwide multicenter survey, participants in the study were not randomly selected from the general population which could have created a bias. However, it is problematic to perform whole spine CT in asymptomatic healthy individuals due to radiologic exposure. Second, this study used a cross-sectional design; therefore, longitudinal studies are necessary to establish causal relationships between cervical spine function and the observed associations. Finally, we did not evaluate cervical spine radiographs in the radiological examination. Therefore, we could not examine the relationship between cervical spine function and cervical ROM and alignment. Further studies are necessary to clarify these issues. Nevertheless, we believe that our findings provide important information that highlights the impact of the spread of ossification on cervical spine function in patients with OPLL.

## Conclusions

The present study revealed the impact of the spread of ossification on cervical spine function, including the impact of sex differences and age-related changes, in a prospective nationwide multicenter study. A higher neck VAS score and a larger number of bridge formations of OPLL in the whole spine were significant predictors of adverse outcomes related to cervical spine function. These findings are important to understand the natural course of OPLL and can serve as a control when evaluating cervical spine function after surgery.

## Supplementary Information

Supplementary Information 1.

Supplementary Information 2.

## Data Availability

The study data and details of materials used may be made available upon reasonable request by sending an e-mail to the first author.
