# Supplementary material for: The impact of ossification spread on cervical spine function in patients with ossification of the posterior longitudinal ligament
Source: Sci Rep. 2021 Jul 12;11:14337. doi: 10.1038/s41598-021-93602-0 (PMC8275804; doi:10.1038/s41598-021-93602-0)
Supplement: Supplementary file 1 — Supplementary Information 1. [file 41598_2021_93602_MOESM1_ESM.docx]

**The impact of ossification spread on cervical spine function in patients with ossification of the posterior longitudinal ligament**

Keiichi Katsumi^1,2,21*^, Takashi Hirai^3,21^, Toshitaka Yoshii^3,21^ , Satoshi Maki^4,21^, Kanji Mori^5,21^, Narihito Nagoshi^6,21^, Soraya Nishimura^6,21^, Kazuhiro Takeuchi^7,21^, Shuta Ushio^3,21^, Takeo Furuya^4,21^, Kei Watanabe^2,21^, Norihiro Nishida^8,21^, Kota Watanabe^6,21^, Takashi Kaito^9,21^, Satoshi Kato^10,21^, Katsuya Nagashima^11,21^, Masao Koda^11,21^, Kenyu Ito^12,21^, Shiro Imagama^12,21^, Yuji Matsuoka^13,21^, Kanichiro Wada^14,21^, Atsushi Kimura^15,21^, Tetsuro Ohba^16,21^, Hiroyuki Katoh^17,21^, Yukihiro Matsuyama^18,21^, Hiroshi Ozawa^19,21^, Hirotaka Haro^16,21^, Katsushi Takeshita^15,21^, Masahiko Watanabe^17,21^, Morio Matsumoto^6,21^, Masaya Nakamura^6,21^, Masashi Yamazaki^11,21^, Atsushi Okawa^3,21^ & Yoshiharu Kawaguchi^20,21^

^1^Spine Center, Department of Orthopedic Surgery, Niigata Central Hospital, 1-18 Shinkocho, Chuo-ku, Niigata, Niigata 950-8556, Japan

^2^Department of Orthopedic Surgery, Niigata University Medical and Dental General Hospital, 1-757 Asahimachidori, Chuo-ku, Niigata, Niigata 951-8510, Japan

^3^Department of Orthopedic Surgery, Tokyo Medical and Dental University, 1-5-45 Yushima, Bunkyo-ku, Tokyo 113-8519, Japan

^4^Department of Orthopaedic Surgery, Chiba University Graduate School of Medicine, 1-8-1 Inohana, Chuo-ku, Chiba, Chiba 260-0856, Japan

^5^Department of Orthopaedic Surgery, Shiga University of Medical Science, Tsukinowa-cho, Seta, Otsu, Shiga 520-2192, Japan

^6^Department of Orthopaedic Surgery, Keio University, School of Medicine, 35 Shinanomachi, Shinjuku-ku, Tokyo 160–8582, Japan

^7^Department of Orthopedic Surgery, National Hospital Organization Okayama Medical Center, 1711–1 Tamasu, Okayama, Okayama 701–1154, Japan

^8^Department of Orthopaedic Surgery, Yamaguchi University Graduate School of Medicine, 1-1-1 Minamikogushi, Ube, Yamaguchi 755-8505, Japan

^9^Department of Orthopaedic Surgery, Osaka University Graduate School of Medicine, 2-2 Yamadaoka, Suita, Osaka 565-0871, Japan

^10^Department of Orthopedic Surgery, Graduate School of Medical Sciences, Kanazawa University, 13-1

Takaramachi, Kanazawa, Ishikawa 920-8641, Japan

^11^Department of Orthopaedic Surgery, Faculty of Medicine, University of Tsukuba, 2-1-1 Amakubo, Tsukuba, Ibaraki 305-8576, Japan

^12^Department of Orthopedic Surgery, Nagoya University Graduate School of Medicine, 65 Tsurumaicho, Showa-ku, Nagoya, Aichi 466-0065, Japan

^13^Department of Orthopedic Surgery, Tokyo Medical University, 6-7-1 Nishishinjuku, Shinjuku-ku, Tokyo 160-0023, Japan

^14^Department of Orthopaedic Surgery, Hirosaki University Graduate School of Medicine, 53 Honcho, Hirosaki, Aomori 036-8203, Japan

^15^Department of Orthopaedic Surgery, Jichi Medical University, 3311-1 Yakushiji, Shimotsuke, Tochigi 329-0498, Japan

^16^Department of Orthopedic Surgery, University of Yamanashi, 1110 Shimokato, Chuo-ku, Yamanashi 409-3898, Japan

^17^Department of Orthopaedic Surgery, Surgical Science, Tokai University School of Medicine, 143 Shimokasuya, Isehara, Kanagawa 259-1143, Japan

^18^Department of Orthopedic Surgery, Hamamatsu University School of Medicine, 1-20-1 Handayama, Hamamatsu, Shizuoka 431-3125, Japan

^19^Department of Orthopaedic Surgery, Tohoku Medical and Pharmaceutical University, 1-12-1 Fukumuro, Miyagino-ku, Sendai, Miyagi 983-8512, Japan

^20^Department of Orthopedic Surgery, Faculty of Medicine, University of Toyama, 2630 Sugitani, Toyama, Toyama 930-0194, Japan

^21^Japanese Organization of the Study for Ossification of Spinal Ligament (JOSL), Tokyo, Japan

**Corresponding Author:**

Keiichi Katsumi, MD, PhD

Spine Center, Department of Orthopedic Surgery, Niigata Central Hospital

1-18 Shinkocho, Chuo-ku, Niigata, Niigata 950-8556, Japan

Phone: +81-25-285-8811; Fax: +81-25-285-4419

E-mail: [kkatsu_os@yahoo.co.jp](mailto:kkatsu_os@yahoo.co.jp)

ORCID ID: 0000-0002-3997-3593

**Supplemental Table 1.** Scoring System for the Japanese Orthopaedic Association score

| **Ⅰ**　Upper extremity motor function  0: Unable to eat unaided with any type of table utensil, including chopsticks, spoon, or fork  and/or unable to fasten buttons of any size  1: Can eat unaided with spoon and/or fork but not chopsticks  2: Either eating with or writing is possible but not practical and/or large buttons can be fastened  3: Either eating with chopsticks or writing is clumsy but possible and/or cuff buttons can be fastened  4: Normal  **Ⅱ**　Lower extremity motor function  0: Unable to stand and walk by any means  0.5: 　Able to stand but unable to walk  1: Unable to walk without a cane or other support on level ground  1.5: Able to walk without support but with a clumsy gait  2: Walks independently on level ground but needs support on stairs  2.5: Walks independently when going upstairs but needs support when going downstairs  3: Capable of walking fast but clumsily  4: 　　 Normal  **Ⅲ**　Sensory function  A. Upper extremities  0: Complete loss of touch and pain sensation  0.5: ≤50% normal sensation and/or severe pain or numbness  1: >60% normal sensation and/or moderate pain or numbness  1.5: Slight subjective numbness without any objective sensory deficit  2: Normal  B. Lower extremities Same as A  C. Trunk Same as A  **Ⅳ**　Bladder function  0: Urinary retention and/or incontinence  1: Sensation of retention, dribbling, thin stream, and/or incomplete continence  2: Urinary retention and/or pollakiuria  3: Normal |
| --- |
| **Total score for normal** = (I + II + III = IV) = 17  **Recovery rate** = (postoperative score minus preoperative score) ×100 / (17 minus preoperative score) |
