# Supplementary material for: The impact of ossification spread on cervical spine function in patients with ossification of the posterior longitudinal ligament
Source: Sci Rep. 2021 Jul 12;11:14337. doi: 10.1038/s41598-021-93602-0 (PMC8275804; doi:10.1038/s41598-021-93602-0)
Supplement: Supplementary file 2 — Supplementary Information 2. [file 41598_2021_93602_MOESM2_ESM.docx]

**The impact of ossification spread on cervical spine function in patients with ossification of the posterior longitudinal ligament**

Keiichi Katsumi^1,2,21*^, Takashi Hirai^3,21^, Toshitaka Yoshii^3,21^ , Satoshi Maki^4,21^, Kanji Mori^5,21^, Narihito Nagoshi^6,21^, Soraya Nishimura^6,21^, Kazuhiro Takeuchi^7,21^, Shuta Ushio^3,21^, Takeo Furuya^4,21^, Kei Watanabe^2,21^, Norihiro Nishida^8,21^, Kota Watanabe^6,21^, Takashi Kaito^9,21^, Satoshi Kato^10,21^, Katsuya Nagashima^11,21^, Masao Koda^11,21^, Kenyu Ito^12,21^, Shiro Imagama^12,21^, Yuji Matsuoka^13,21^, Kanichiro Wada^14,21^, Atsushi Kimura^15,21^, Tetsuro Ohba^16,21^, Hiroyuki Katoh^17,21^, Yukihiro Matsuyama^18,21^, Hiroshi Ozawa^19,21^, Hirotaka Haro^16,21^, Katsushi Takeshita^15,21^, Masahiko Watanabe^17,21^, Morio Matsumoto^6,21^, Masaya Nakamura^6,21^, Masashi Yamazaki^11,21^, Atsushi Okawa^3,21^ & Yoshiharu Kawaguchi^20,21^

^1^Spine Center, Department of Orthopedic Surgery, Niigata Central Hospital, 1-18 Shinkocho, Chuo-ku, Niigata, Niigata 950-8556, Japan

^2^Department of Orthopedic Surgery, Niigata University Medical and Dental General Hospital, 1-757 Asahimachidori, Chuo-ku, Niigata, Niigata 951-8510, Japan

^3^Department of Orthopedic Surgery, Tokyo Medical and Dental University, 1-5-45 Yushima, Bunkyo-ku, Tokyo 113-8519, Japan

^4^Department of Orthopaedic Surgery, Chiba University Graduate School of Medicine, 1-8-1 Inohana, Chuo-ku, Chiba, Chiba 260-0856, Japan

^5^Department of Orthopaedic Surgery, Shiga University of Medical Science, Tsukinowa-cho, Seta, Otsu, Shiga 520-2192, Japan

^6^Department of Orthopaedic Surgery, Keio University, School of Medicine, 35 Shinanomachi, Shinjuku-ku, Tokyo 160–8582, Japan

^7^Department of Orthopedic Surgery, National Hospital Organization Okayama Medical Center, 1711–1 Tamasu, Okayama, Okayama 701–1154, Japan

^8^Department of Orthopaedic Surgery, Yamaguchi University Graduate School of Medicine, 1-1-1 Minamikogushi, Ube, Yamaguchi 755-8505, Japan

^9^Department of Orthopaedic Surgery, Osaka University Graduate School of Medicine, 2-2 Yamadaoka, Suita, Osaka 565-0871, Japan

^10^Department of Orthopedic Surgery, Graduate School of Medical Sciences, Kanazawa University, 13-1

Takaramachi, Kanazawa, Ishikawa 920-8641, Japan

^11^Department of Orthopaedic Surgery, Faculty of Medicine, University of Tsukuba, 2-1-1 Amakubo, Tsukuba, Ibaraki 305-8576, Japan

^12^Department of Orthopedic Surgery, Nagoya University Graduate School of Medicine, 65 Tsurumaicho, Showa-ku, Nagoya, Aichi 466-0065, Japan

^13^Department of Orthopedic Surgery, Tokyo Medical University, 6-7-1 Nishishinjuku, Shinjuku-ku, Tokyo 160-0023, Japan

^14^Department of Orthopaedic Surgery, Hirosaki University Graduate School of Medicine, 53 Honcho, Hirosaki, Aomori 036-8203, Japan

^15^Department of Orthopaedic Surgery, Jichi Medical University, 3311-1 Yakushiji, Shimotsuke, Tochigi 329-0498, Japan

^16^Department of Orthopedic Surgery, University of Yamanashi, 1110 Shimokato, Chuo-ku, Yamanashi 409-3898, Japan

^17^Department of Orthopaedic Surgery, Surgical Science, Tokai University School of Medicine, 143 Shimokasuya, Isehara, Kanagawa 259-1143, Japan

^18^Department of Orthopedic Surgery, Hamamatsu University School of Medicine, 1-20-1 Handayama, Hamamatsu, Shizuoka 431-3125, Japan

^19^Department of Orthopaedic Surgery, Tohoku Medical and Pharmaceutical University, 1-12-1 Fukumuro, Miyagino-ku, Sendai, Miyagi 983-8512, Japan

^20^Department of Orthopedic Surgery, Faculty of Medicine, University of Toyama, 2630 Sugitani, Toyama, Toyama 930-0194, Japan

^21^Japanese Organization of the Study for Ossification of Spinal Ligament (JOSL), Tokyo, Japan

**Corresponding Author:**

Keiichi Katsumi, MD, PhD

Spine Center, Department of Orthopedic Surgery, Niigata Central Hospital

1-18 Shinkocho, Chuo-ku, Niigata, Niigata 950-8556, Japan

Phone: +81-25-285-8811; Fax: +81-25-285-4419

E-mail: [kkatsu_os@yahoo.co.jp](mailto:kkatsu_os@yahoo.co.jp)

ORCID ID: 0000-0002-3997-3593

**Supplemental Table 2.** Japanese Orthopaedic Association Cervical Myelopathy Evaluation Questionnaire.

With regard to your state of health during the last week, please circle the number of the answer for each of the following questions that best applies. If your condition varies from day to day or throughout the day, please circle the number when that item is at its worst.

**Q1-1 While in the sitting position, can you look up at the ceiling by tilting your head upward?**

1) Impossible. 2) Possible to some degree (with effort). 3) Possible without difficulty.

**Q1-2 Can you drink a glass of water without stopping despite your neck symptoms?**

1) Impossible. 2) Possible to some degree. 3) Possible without difficulty.

**Q1-3 While in the sitting position, can you turn your head toward a person who is seated to the side but behind you and speak to that person while looking at his/her face?**

1) Impossible. 2) Possible to some degree. 3) Possible without difficulty.

**Q1-4 Can you look at your feet when you go down stairs?**

1) Impossible. 2) Possible to some degree. 3) Possible without difficulty.

**Q2-1 Can you fasten the front buttons of your blouse or shirt with both hands?**

1) Impossible. 2) Possible if I take my time. 3) Possible without difficulty.

**Q2-2 Can you eat a meal with your dominant hand using a spoon or a fork?**

1) Impossible. 2) Possible if I take my time. 3) Possible without difficulty.

**Q2-3 Can you raise your arm? (Answer for the weaker side.)**

1) Impossible. 2) Possible up to shoulder level. 3) Possible although the elbow and/or wrist is slightly flexed. 4) I can raise it straight upward.

**Q3-1 Can you walk on a flat surface?**

1) Impossible. 2) Possible but slowly even with support. 3) Possible only with the support of a handrail, cane, or walker. 4) Possible but slowly without any support. 5) Possible without difficulty.

**Q3-2 Can you stand on either leg without the support of your hand? (without needing to support yourself)**

1) Impossible with either leg. 2) Possible on either leg for more than ten seconds. 3) Possible for each leg individually for more than ten seconds.

**Q3-3 Do you have difficulty going up stairs?**

1) I have great difficulty. 2) I have some difficulty. 3) I have no difficulty.

**Q3-4 Do you have difficulty in one of the following movements: bending forward, Kneeling, or stooping?**

1) I have great difficulty. 2) I have some difficulty. 3) I have no difficulty.

**Q3-5 Do you have difficulty walking more than 15 minutes?**

1) I have great difficulty. 2) I have some difficulty. 3) I have no difficulty.

**Q4-1 Do you have urinary incontinence?**

1) Always. 2) Frequently. 3) Only when I have not passed urine for more than 2 hours. 4) When sneezing or straining. 5) No.

**Q4-2 How often do you go to the bathroom at night?**

1) Three times or more. 2) Once or twice. 3) Rarely.

**Q4-3 Do you have a feeling of residual urine in your bladder after voiding?**

1) Most of the time. 2) Sometimes. 3) Rarely.

**Q4-4 Can you start your urine stream immediately when you want to void?**

1) Usually not. 2) Sometimes. 3) Most of the time.

**Q5-1 How do you rate your present health condition?**

1) Poor. 2) Fair. 3) Good. 4) Very good. 5) Excellent.

**Q5-2 Have you been unable to work or go about your normal activities?**

1) I have not been able to do them at all. 2) I have been unable to do them most of the time. 3) I have sometimes been unable to do them. 4) I have been able to do them most of the time. 5) I have always been able to do them.

**Q5-3 Has your work routine been hindered because of pain?**

1) Greatly. 2) Moderately. 3) Slightly (somewhat). 4) Little (minimally). 5) Not at all.

**Q5-4 Have you been feeling discouraged or depressed?**

1) Always. 2) Frequently. 3) Sometimes. 4) Rarely. 5) Never.

**Q5-5 Do you ever feel exhausted?**

1) Always. 2) Frequently. 3) Sometimes. 4) Rarely. 5) Never.

**Q5-6 Do you feel happy?**

1) Never. 2) Rarely. 3) Sometimes. 4) Almost always. 5) Always.

**Q5-7 Do you think you are in decent health?**

1) Not at all (my health is very poor). 2) Barely (my health is poor). 3) Not much (my health is average). 4) Fairly (my health is better than average). 5) Yes (I am healthy).

**Q5-8 Do you feel your health will get worse?**

1) Very much so. 2) A little bit at a time. 3) Sometimes yes and sometimes no. 4) Not very much. 5) Not at all.
